# Subacute Changes in *N*-Acetylaspartate (NAA) Following Ischemic Stroke: A Serial MR Spectroscopy Pilot Study

**DOI:** 10.3390/diagnostics10070482

**Published:** 2020-07-16

**Authors:** Ndaba Mazibuko, Ruth O’Gorman Tuura, Laszlo Sztriha, Owen O’Daly, Gareth J. Barker, Steven C. R. Williams, Michael O’Sullivan, Lalit Kalra

**Affiliations:** 1 Department of Basic and Clinical Neuroscience, Institute of Psychiatry, Psychology and Neuroscience (IoPPN), King’s College London, London SE5 8AF, UK; ndabezinhle.mazibuko@kcl.ac.uk (N.M.); laszlo.sztriha@kcl.ac.uk (L.S.); lkal792@aucklanduni.ac.nz (L.K.); 2 Department of Neuroimaging, Institute of Psychiatry, Psychology and Neuroscience, King’s College London, London SE5 8AF, UKgareth.barker@kcl.ac.uk (G.J.B.); steve.williams@kcl.ac.uk (S.C.R.W.); m.osullivan1@uq.edu.au (M.O.); 3 Center for MR Research, Children’s Hospital, Zürich, Steinwiesenstrasse 75, CH-8032 Zurich, Switzerland; 4 Department of Neurology, King’s College Hospital NHS Foundation Trust, London SE5 9RS, UK; 5 UQ Centre for Clinical Research, University of Queensland, Herston, QLD 4029, Australia

**Keywords:** ischaemic stroke, subacute, inflammation, recovery, *N*-acetylaspartate (NAA), MR spectroscopy

## Abstract

Preservation of neuronal tissue is crucial for recovery after stroke, but studies suggest that prolonged neuronal loss occurs following acute ischaemia. This study assessed the temporal pattern of neuronal loss in subacute ischemic stroke patients using ^1^H magnetic resonance spectroscopy, in parallel with functional recovery at 2, 6 and 12 weeks after stroke. Specifically, we measured *N*-acetylaspartate (NAA), choline, myoinositol, creatine and lactate concentrations in the ipsilesional and contralesional thalamus of 15 first-ever acute ischaemic stroke patients and 15 control participants and correlated MRS concentrations with motor recovery, measured at 12 weeks using the Fugl–Meyer scale. NAA in the ipsilesional thalamus fell significantly between 2 and 12 weeks (10.0 to 7.97 mmol/L, *p* = 0.003), while choline, myoinositol and lactate concentrations increased (*p* = 0.025, *p* = 0.031, *p* = 0.001, respectively). Higher NAA concentrations in the ipsilesional thalamus at 2 and 12 weeks correlated with higher Fugl Meyer scores at 12 weeks (*p* = 0.004 and *p* = 0.006, respectively). While these results should be considered preliminary given the modest sample size, the progressive fall in NAA and late increases in choline, myoinositol and lactate may indicate progressive non-ischaemic neuronal loss, metabolically depressed neurons and/or diaschisis effects, which have a detrimental effect on motor recovery. Interventions that can potentially limit this ongoing subacute tissue damage may improve stroke recovery.

## 1. Introduction

Stroke remains the leading cause of acquired adult disability, with up to 30% of survivors suffering from permanent disability [1]. Preservation of neuronal tissue after injury is key to recovery and is dependent upon several processes in the post-infarction phase, including further ischaemic damage, reperfusion, inflammation, gliosis and neuronal repair [2,3]. These processes take place not only in the core of the infarct but also in structurally and functionally connected areas, remote from the infarct site.

Neuroimaging methods like Magnetic Resonance (MR) imaging may facilitate the investigation of these processes and their relationship to functional recovery [4]. Previous diffusion and spectroscopic magnetic resonance imaging studies have demonstrated progressive neuronal loss in the ipsilesional thalamus of patients with middle cerebral artery infarction up to 90 days after stroke [5,6,7], while PET studies have shown late microglial and tissue macrophage activation in intact regions connected to the core infarct that can destroy tissue and hamper recovery [8].

In addition, many cellular elements involved in these processes have distinctive chemical signatures, which can be measured with Proton Magnetic Resonance Spectroscopy (1H-MRS) [9,10]. Specifically, 1H-MRS is sensitive to changes in a number of neuro-metabolites, including *N*-acetylaspartate (NAA), which is synthesised exclusively in mitochondria of normal neurons and which has been related to the proportion or patency of normal neurons [11]. Additional neuro-metabolites detectable with 1H-MRS include Choline (Cho) containing compounds, which are involved in membrane synthesis and degradation and are elevated in high turnover states [10], and myoinositol (mI), predominantly reflecting increased glial activity [10]. A rapid rise in lactate indicates a switch from oxidative metabolism to anaerobic glycolysis with ischaemia, but a sustained low rise has been attributed to anaerobic glycolysis in activated macrophages [12]. Creatine (Cr) is a marker for energy metabolism and reflects the balance between energy demand and supply [10]. In addition, these post-infarct processes are associated with changes in perfusion [13], which can be reliably measured using Arterial Spin Labelling (ASL) in acute stroke patients [14].

Previous studies of NAA after stroke confirm an acute decrease in NAA in the centre of ischemic lesions consistent with neuronal loss [6,15]. Although much of the total tissue damage occurs in the first few hours after stroke, there is evidence that damage may continue subacutely from secondary factors [7,8]. There are also ongoing processes of repair; animal studies demonstrate neurogenesis in the periventricular subependymal regions with neuron migration towards damaged areas, which may increase neuron density [3]. The duration of further damage or repair, the factors that influence these processes, and their relationship to functional recovery remain unclear but may offer potential targets for therapeutic interventions.

The aim of this study was to investigate the time course of subacute neuronal loss in a structurally undamaged region connected to, but not included in, the infarct using ^1^H-MRS signals as markers of cellular changes. The relationship between perfusion (quantified with arterial spin labelling), neuronal density as measured by NAA concentrations at 2 and 12 weeks and functional recovery at 12 weeks was also assessed, in order to test the hypothesis that MRI and MRS markers of cellular changes may represent predictors for functional outcome after stroke.

## 2. Materials and Methods

### 2.1. Subjects

**Figure 1:**
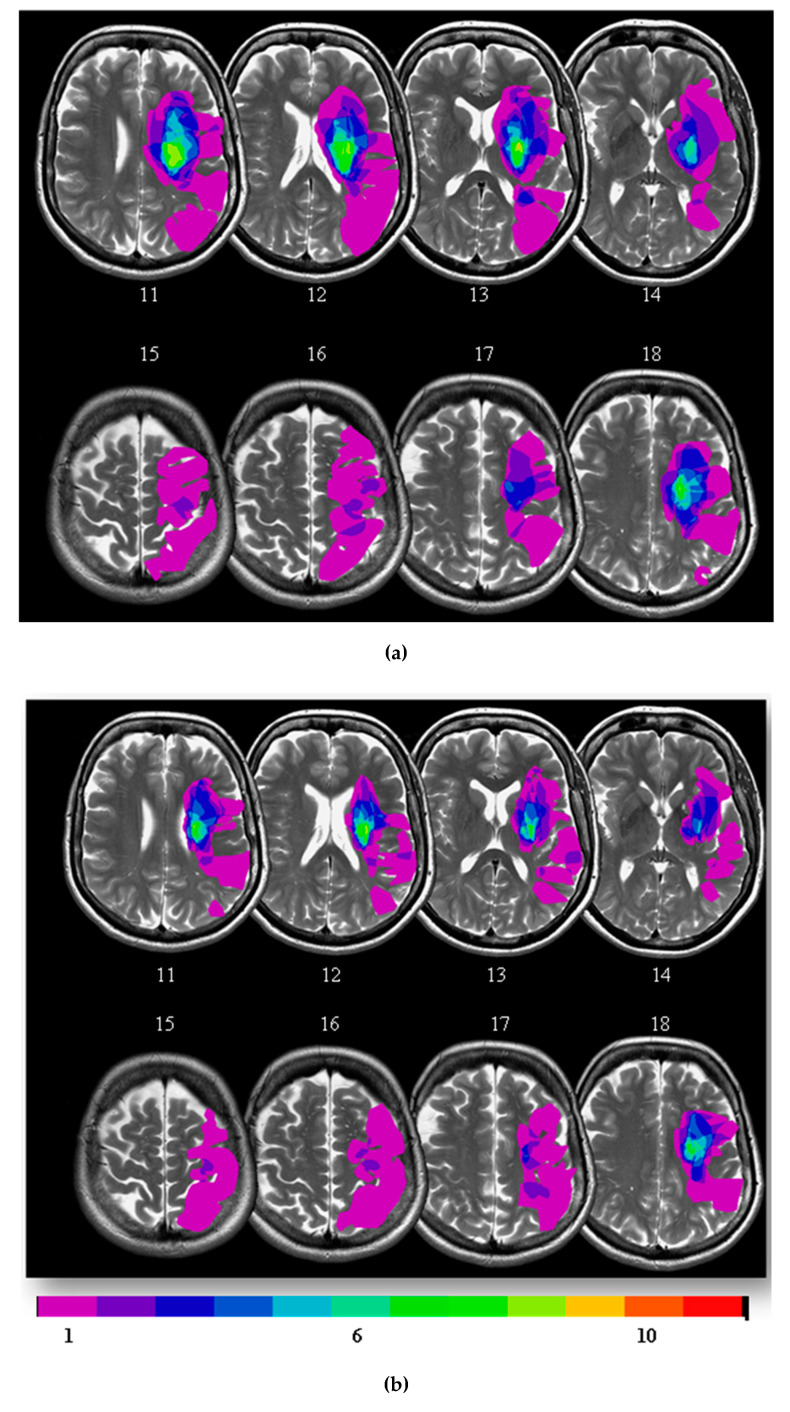
T2 weighted lesion overlay maps of 15 stroke patients at baseline (**a**) and 12 weeks (**b**). For patients with right-sided strokes, images were flipped before delineating the lesions.

The study included 23 right-handed patients aged 18–75 years with moderate to severe cortical ischemic stroke recruited within 2 weeks of stroke onset and 15 age and sex matched controls. Inclusion criteria were first-ever ischaemic strokes on MR imaging, cortical lesion in the middle cerebral artery territory area with thalamic sparing (Figure 1), National Institutes of Health Stroke Scale (NIHSS) Score 2–3 for motor arm and leg, and capacity to consent and participate with imaging and clinical assessments. Patients with significant (≥50%) carotid or intracranial artery stenosis, age-related white matter changes (ARWMC) score ≥2 in the white matter or basal ganglia [16] or contraindications to MR imaging were excluded. Age and sex matched controls with no evidence of silent infarction on MR imaging or other exclusion criteria (e.g., contraindications for MRI) were recruited by invitation from the community. Stroke patients received inpatient or outpatient rehabilitation therapy as part of routine care. Informed consent was obtained, and the study was approved by the London South East Research Ethics Committee (09/H0803/149).

### 2.2. Imaging Techniques

MR imaging studies were performed with a 3.0 Tesla General Electric HDx MRI scanner (GE Medical Systems, Milwaukee, WI, USA), using an 8-channel head coil. The imaging protocol lasted about 60 min and consisted of T1 and T2 weighted structural imaging (15 min), Diffusion Tensor Imaging (15 min), MRS (20 min) and ASL (5 min).

Structural imaging for anatomical localisation and assessment of volume of infarction was performed using T2-weighted and fluid attenuation inversion recovery (FLAIR) sequences, prescribed parallel to the AC/PC line. T2-weighted scans had echo time (TE) = 103 ms, repetition time (TR) = 3520 ms, 19 slices (5 mm/2 mm gap); FLAIR scans had inversion time (TI) = 2000 ms, echo time (TE) = 128 ms, repetition time (TR) = 2000 ms, 36 slices (4mm/4mm gap). Parallel imaging (ASSET, with a speed up factor of 2) was used to allow greater slice coverage per unit time. Lesion overlap maps were generated after manually outlining the lesions on the T2-weighted images for each patient in mricron (http://neuro.debian.net/pkgs/mricron.html).

**Figure 2:**
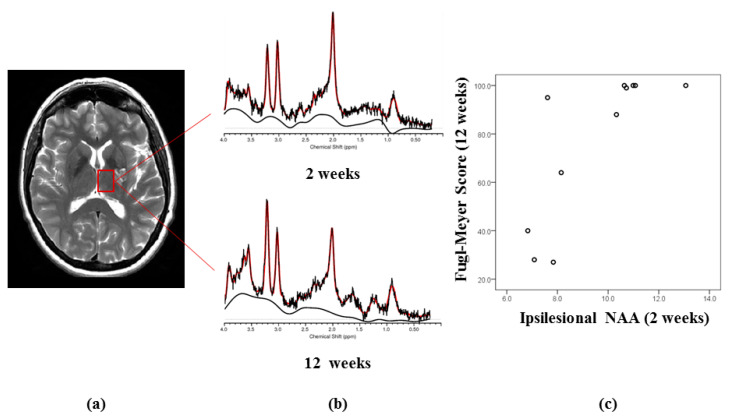
(**a**) Volume of Interest (VOI) for 1H-MRS, (**b**) representative MRS spectra acquired from a stroke patient at baseline and 12 weeks, (**c**) Scatter plot depicting the Fugl–Meyer score at 12 weeks vs. ipsilesional (thalamic) *N*-acetylaspartate (NAA) at 2 weeks.

The MRS volume of interest (VOI) was centred on the ipsilesional thalamus leaving a clear margin with affected areas (Figure 2). The thalamus is a fixed landmark between the sub-ependymal zone and infarcted tissue, where post-ischaemic cellular/metabolic events have been described in experimental models [3]. In addition, it is a major relay for centrifugal cortical pathways, known to show neuronal loss and inflammation after stroke [5,7,8]. The contralesional thalamus was also studied because of evidence of activation during recovery in functional imaging studies [17].

MRS studies were localised using a 3D fast inversion-recovery prepared spoiled gradient acquisition in the steady state (IR-SPGR) scan, with TI = 450 ms, TE = 3 ms and TR = 7 ms. The volumetric IR-SPGR images were segmented into grey matter, white matter and CSF maps using SPM (www.fil.ion.ucl.ac.uk/spm). Single voxel 1HMR spectra were acquired from thalamic voxels of interest (VOI, size: 20 × 15 × 20 mm^3^), using a point resolved spectroscopy (PRESS) sequence with TE = 30 ms and TR = 3 s. Water-scaled metabolite concentrations were derived with LC Model (Figure 2) [18]. Estimated concentrations with a Cramer Rao Lower Bound (%CRLB) of greater than 20% for NAA, Cr, Cho and mI, or greater than 40% for Lactate were excluded from further statistical analyses. The in vivo water-scaled concentrations reported by LC Model were divided by the SPM-derived fractional content of brain tissue (p[GM] + p[WM], where p[GM] and p[WM] represent the percentage of grey matter and white matter in the voxel, respectively) to correct for partial volume contamination of cerebrospinal fluid (CSF) in the ^1^H-MRS voxel. The water concentration used for water scaling was also corrected for the amount of CSF in the ^1^H-MRS voxel, assuming a CSF water concentration of 55.556 M.

**Figure 3:**
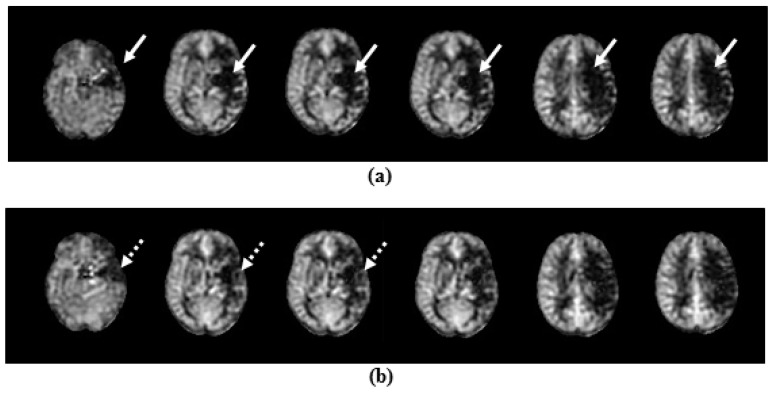
Representative Arterial Spin Labelling (ASL) dataset from a stroke patient at 2 weeks (**a**) and 12 weeks (**b**). A large area of reduced perfusion is seen at 12 weeks within the area affected by the stroke (solid arrows), but some regions within the affected area show a mild recovery of perfusion at 12 weeks (dashed arrows).

Whole-brain resting cerebral perfusion images were collected with a background-suppressed, pulsed continuous arterial spin labelling (pCASL) sequence, using a 3D stack of spirals fast spin echo readout [19]. Sixty axial slices were collected, with TR = 5.5 s, TE = 25 ms, acquisition matrix = 64 × 64 pixels, slice thickness = 3 mm, field of view = 24 cm, and reconstruction matrix = 128 × 128 pixels, resulting in an effective voxel size of 1.9 × 1.9 × 3 mm^3^ (see Figure 3 for a representative pCASL dataset).

Baseline scanning was performed at 2 weeks post-stroke to reduce confounds from vascular or chemical changes from impaired autoregulation, acute ischemic cascade, hypoxic lactate surge and cerebral oedema. Follow-up assessments were undertaken at 6 ± 1 and 12 ± 3 weeks after stroke, the time-points associated with greatest functional recovery in stroke rehabilitation studies [17]. The patient’s head position during the initial scan was recorded and replicated on follow-up scans. The spectroscopy VOI was carefully placed in the same position relative to the lesion and relevant anatomical landmarks on each occasion.

### 2.3. Clinical Assessments

Baseline assessment at 2 weeks included age, sex, vascular risk profile, medication and NIHSS score. Motor impairment was assessed using the Fugl–Meyer scale [20] and repeated at 6 and 12 weeks. Overall function was measured using the Functional Independence Measure (FIM) at the same time points [21]. Motor assessments were performed by trained therapists following a standardised protocol. Clinical assessments were undertaken on the same day as MR imaging, but investigators undertaking image analysis were blinded to the findings of the clinical assessments.

### 2.4. Data Analysis

For each patient, we calculated values of CBF, NAA, Cho, mI, Cr and lactate for each VOI. Data from ipsi- and contra-lateral thalamus for all patients and controls were normally distributed (Kolmogorov–Smirnov tests). Differences in CBF and metabolites between patients and controls were assessed by independent sample *t* tests.

Within-subject differences in CBF and metabolites between baseline, 6 and 12 weeks were assessed using ANCOVA for repeated measures adjusted for age, baseline NIHSS score and lesion volume, using predictive model-based imputation for missing data points (5 in 120 assessments). This method can assess within-subject differences whilst allowing for between subject variations and reduces bias due to only including patients in whom all data for the 3 time points is available [22].

Spearman rank order correlation was used to evaluate hypothesis driven relationships between NAA, CBF and Fugl–Meyer score at 12 weeks. In order to minimise false positive results because of multiple testing, correlations were only performed if NAA concentration or CBF was significantly different from controls at baseline or showed a significant temporal trend. Differences were considered significant at the two-sided *p* value of *p* ≤ 0.05.

## 3. Results

### 3.1. Patient Characteristics

Of the 23 stroke patients recruited at baseline, 4 declined further imaging, and 4 were withdrawn because of poor image quality. The mean age of the remaining 15 patients (9 men) was 51.4 ± 13.3 years (Range 21–75), and their median National Institutes of Health Stroke Scale (NIHSS) score was 9 (IQR 3–15) at 2 weeks. Of these, 3 had a total anterior circulation stroke syndrome, and 12 had a partial anterior circulation stroke syndrome (Figure 1). Vascular risk factors in stroke patients included hypertension (5/15), type II diabetes mellitus (3/15), hypercholesterolaemia (8/15) and smoking (4/15). The mean age of healthy controls (10 men) was 49.1 ± 9.3 years. Vascular risk factors in controls included hypertension (6/15), type II diabetes mellitus (1/15), hypercholesterolaemia (4/15) and smoking (4/15).

### 3.2. Temporal Evolution of NAA and Other Metabolites

Twelve of the fifteen infarcts were in the left hemisphere, and the median stroke lesion volume at 2 weeks was 20.4 (IQR 6.7–37.4) cm^3^. The median infarct volume was 11.6 (IQR 7.1–23.0) cm^3^ at 12 weeks.

Metabolite concentrations from MRS voxels positioned in the ipsilesional and contralesional thalamus are given in Table 1, together with the statistical results from both the repeated-measures ANCOVA assessing longitudinal changes in the patient group, and the two-sample *t*-test assessing groupwise differences between the controls and the patients at baseline.

The NAA concentration in the structurally intact ipsilesional thalamus was comparable to that measured in the healthy controls at 2 weeks. There was a significant decrease thereafter, reaching a nadir at 6 weeks followed by a small increase by 12 weeks (Table 1). The repeated measures ANCOVA model showed that NAA concentration was significantly reduced between 2 and 12 weeks (*p* = 0.003, Table 1). These results remained significant after removal of the patient with the largest lesion volume.

Choline concentration in the intact ipsilesional thalamus, indicative of high membrane turnover, was significantly higher than in controls at 2 weeks (*p* = 0.009, Table 1) and continued to rise between 2–12 weeks (*p* = 0.025). Myoinisitol concentration, a measure of glial activity, was comparable with controls at 2 weeks but increased significantly between 2–12 weeks (*p* = 0.031).

Thalamic lactate concentration was comparable with controls at baseline but increased significantly by 12 weeks. The concentration of creatinine, a marker for energy homeostasis, was significantly lower in the ipsilesional thalamus compared with healthy controls at 2 weeks (Table 1) but increased significantly between 2 and 12 weeks (*p* = 0.003)

NAA, Cho, mI, Lac and Cr concentrations in the contralesional thalamus were comparable with healthy controls at 2 weeks, and there were no significant changes in their concentrations between 2 and 12 weeks.

### 3.3. Temporal Evolution of CBF

Ipsilesional thalamic perfusion was lower compared with healthy controls and also to the contralesional thalamus at 2 weeks (Table 1) but increased significantly between 2 and 12 weeks (*p* = 0.004), while still remaining below control values. CBF in the contralesional thalamus was comparable to that in the healthy controls at 2 weeks and showed no significant temporal changes.

### 3.4. Relationship of MRS and ASL Measures with Outcome

Stroke patients showed significant clinical improvements in neurological deficits and motor impairments between 2 and 12 weeks. The mean Fugl–Meyer motor score improved from 43.3 ± 30.0 (SD) at 2 weeks to 78.3 ± 30.1 (*p* = 0.009) at 12 weeks (score range 0–100, worst to best), and their FIM score, a measure of functional ability, improved from 65 ± 19 to 89 ± 5 (*p* = 0.003, score range 18–126, worst to best).

Higher NAA concentration in the ipsilesional thalamus at 2 and 12 weeks correlated with a higher Fugl–Meyer score at 12 weeks (Spearman’s *rho* = 0.79, *p* = 0.004 and *rho* = 0.80, *p* = 0.006, respectively). Higher choline concentration at 12 weeks correlated with lower Fugl–Meyer scores at 12 weeks (*rho* = −0.756, *p* = 0.018).

Higher CBF in the ipsilesional thalamus at 2 weeks correlated with higher NAA concentration at 12 weeks (*r*^2^ = 0.42, *p* = 0.002) and higher Fugl–Meyer score at 12 weeks (*r*^2^ = 0.817, *p* = 0.001).

## 4. Discussion

The longitudinal changes observed in NAA and CBF between 2 and 12 weeks after stroke provide an insight into subacute neuronal loss after ischaemic stroke and its relationship to recovery. They show that in the ipsilesional, structurally intact thalamus there is further neuronal loss (NAA reduction) after 2 weeks of acute injury, which reaches maximum at approximately 6 weeks, followed by a rebound at 12 weeks to values lower than at baseline. They also demonstrate a relationship between surviving and functioning neurons (NAA concentration) and extent of motor recovery following stroke, such that higher ipsilesional thalamic NAA levels seem to be linked to a better clinical recovery. Conversely, elevated Choline levels were predictive of a worse clinical recovery, while there was a suggestion that higher regional perfusion may be important in limiting neuronal loss and promoting motor recovery.

A possible explanation for the decrease in NAA concentrations at 6 weeks and a rebound at 12 weeks could be experimental bias due to changes in head or voxel positioning, scanner drift, partial volume averaging, changes in metabolite relaxation times or tissue oedema. Extreme care was taken to match position of voxels on all scans and standardise imaging protocols. Changes in NAA concentration are unlikely to be due to dilution effects of residual tissue oedema because the voxels were placed in a region outside the infarct zone, and baseline imaging was performed at 2 weeks, by which time tissue oedema would be resolved [23]. That the changes in NAA concentrations genuinely represent a loss of density or function of neurons is supported by increases in choline and myoinositol concentrations, suggestive of increased membrane turnover and gliosis [10] and a lack of similar changes in the contralesional thalamus, where metabolite concentrations remained comparable with controls. It is also unlikely that this decrease in NAA is due to acute ischaemia because the lactate concentrations at two weeks, which would reflect underlying anaerobic glycolysis, were not elevated and were comparable with those in the contralesional thalamus and with healthy controls. The presence of lactate in healthy controls was unexpected, but studies have shown small lactate signals in healthy people, which are considered intrinsic to ageing [24].

Ischemic neuronal damage occurs within the first few hours of stroke. The substantial reductions in NAA beyond 12–14 days and in areas remote from infarction seen in this and other studies [5,6,7] suggest a loss of neurons (or reduction in neuronal function) beyond the acute ischaemic phase. However, it is quite possible that a wider zone of subacute ischaemia may exist beyond acute injury, and diaschisis may also provide an alternative explanation for the progressive decrease in NAA. In comparison to a previous serial MR spectroscopy study in stroke patients [6], NAA levels reached a nadir slightly later than previously reported (namely at 6 weeks in the present study, rather than at 2 weeks, as reported previously) [6]. However, the nadir of 2 weeks was reported for voxels classified as “possibly or definitely abnormal” based on imaging findings, suggesting that neuronal loss may take place more quickly in the infarct zone than in areas remote from the infarct. Perfusion of the ipsilesional thalamus at baseline was also reduced compared with the contralesional thalamus and controls. While this reduction to 22.7 mL/100 g/min is not severe enough to lead to neuronal death [14], since the perfusion was averaged over the thalamus, there may be areas with this region demonstrating perfusion below the ischemic threshold.

One possible explanation for the apparent loss of neuronal density and/or function may be inflammation due to microglial activation. This is supported by the small rise in lactate indicative of infiltration with macrophages (high rate of anaerobic glycolysis) [12] and increased myoinositol and creatine concentrations suggestive of microglial activation (glial cells have 2–4 times more myoinositol and creatine than neurons [10]. The shared cellular lineage and reaction profiles of microglia and tissue macrophages suggest a neuroinflammatory reaction rather than recruitment of blood-borne cells. Inflammation and neuronal loss have also been reported in previous studies [8,25]. Stroke patients followed up for 150 days showed a late and prolonged microglial response in intact but structurally connected areas, which was dominated by tissue phagocytes and tissue destruction [8]. More recently, experimental stroke studies have shown inflammation 4–8 weeks after lesion induction [25]. Tissue infiltrated with phagocytes showed severe injury and necrosis, whereas tissue with non-phagocytic inflammation remained viable and remodelled after weeks. Non-phagocytic inflammation with remodelling may explain the rebound in NAA seen in this study. Reductions in NAA, which later recover, have also been observed in animal models of multiple sclerosis and attributed to tissue fluid changes secondary to inflammation [26,27].

However, an alternative explanation for the reduction in NAA over time may be diaschisis due to loss of input from infarcted regions. Diaschisis may also underly the apparent reduction in thalamic perfusion, as reduced perfusion due to diaschisis in stroke patients has previously been observed with ASL [28]. Diaschisis has also been reported to underly changes in NAA levels in stroke patients, and recovery of diaschitic tissue has been implicated as an important factor governing functional recovery [29,30].

A relationship between higher ispilesional NAA concentrations in the peri-infarct zone and greater extent of motor recovery has previously been shown in stroke patients studied more than 6 months after stroke [31]. This study extends these findings to intact ipsilesional regions connected to the core infarct in the subacute phase of stroke. It suggests that neuronal degeneration, possibly related to diaschisis or inflammation along the neuronal pathways expected to develop Wallerian degeneration, has a detrimental effect on motor recovery. It also showed that higher ipsilesional perfusion was associated with a significantly lower fall in NAA concentrations between 2 and 12 weeks and higher concentrations of NAA and better motor recovery at 12 weeks. Increased perfusion after stroke has been related to higher NAA concentrations in damaged areas in other studies and has been suggested to be a substrate for recovery [32], but this relationship and the mechanisms underlying it need further exploration.

The strengths of the present study are three fixed and conceptually valid time points for assessment of recovery [17], the evaluation of relevant clinical outcome measures as well as spectroscopic markers, inclusion of all patient data (not just those scanned at all times) by using predictive model-based imputation, extreme care to match position of voxels on all scans, image analysis techniques that minimised bias due to tissue compartments, lesion anatomy or volume loss and standardised measurement of metabolite values. Most previous studies have used either single modality or focused on single metabolites, but few have combined multimodality and multimetabolite measurements in a single investigation. In addition, there are very few spectroscopic studies of changes in spared but functionally connected ipsilesional structures during the phase of greatest motor recovery in stroke patients. As far as we are aware, the present study is the first to link multimodality measurements to clinical outcome measures, enabling the relevance of MRS and MRI measures as predictors of clinical recovery to be assessed.

A major limitation of this investigation is the modest sample size, and corresponding susceptibility to inadvertent bias because of multiple comparisons in a small number of patients. To reduce heterogeneity and potential sources of bias we have used restrictive inclusion criteria, standardised protocols for imaging and performed hypothesis driven analyses. We were unable acquire all data as planned (4/23 withdrew and 4/23 were excluded due to poor data quality, 5 in 120 assessments were not performed) or adjust fully for regional tissue distortion on follow-up scans due to changes in mass effect of the infarct. This effect is likely to be minimal, as patients were included after any oedema would have resolved, and voxels were placed in structurally intact tissue, with a clear margin from the infarct. While the timing of the baseline measurement was selected to avoid confounds from oedema, measurements at an earlier time point would provide additional information, and caution needs to be exercised in extrapolating metabolite findings to underlying histological changes. The metabolic findings of this study are consistent with other longitudinal stroke studies [5,6], histopathology correlates of spectral changes in stroke patients [12] and metabolic profiles of various cell types in in-vitro studies [9,10].

## 5. Conclusions

To conclude, this study provides preliminary spectroscopic evidence of progressive neuronal degeneration in the intact ipsilesional thalamus during the subacute phase of stroke, which correlated inversely with the extent of recovery. Possible mechanisms for post-ischaemic degenerative changes include diaschisis or inflammation along neuronal pathways connected to the core infarct, raising the possibility of targeting this mechanism as a potential therapeutic intervention to improve recovery from stroke.

## Figures and Tables

**Table 1:** Longitudinal changes in the mean concentration of metabolites (measured as mmol/L) and regional perfusion in the intact ipsilesional and contralesional thalamus between 2 and 12 weeks post-stroke.

Metabolite	Group	VOI Location	Baseline	6 Weeks	12 Weeks	*p*-Value (Longitudinal Change in Patients)	*p*-Value (Patients at Baseline vs. Controls) ^§^
NAA (mmol/L)	Patient	Ipsilesional thalamus	10.0 (2.03)	6.85 (4.08)	7.97 (1.69)	0.003 **	
		Contralesional thalamus	10.46 (1.94)	12.18 (3.42)	10.52 (0.92)	0.19	
	Control	Left thalamus	11.17 (1.01)				0.27
		Right thalamus	11.19 (0.59)				0.23
Cho (mmol/L)	Patient	Ipsilesional	2.33 (0.35)	2.51 (1.17)	2.82 (0.58)	0.025 *	
		Contralesional	1.98 (0.28)	2.40 (0.90)	2.56 (1.18)	0.22	
	Control	left	2.06 (0.33)				0.009 **
		right	2.00 (0.21)				0.87
mI (mmol/L)	Patient	Ipsilesional	4.29 (2.06)	4.07 (3.11)	6.33 (3.21)	0.031 *	
		Contralesional	4.38 (2.25)	4.29 (1.54)	4.32 (1.43)	0.82	
	Control	left	4.07 (1.44)				0.62
		right	3.97 (1.06)				0.27
Creatinine (mmol/L)	Patient	Ipsilesional	5.63 (0.36)	6.31 (1.33)	7.38 (1.75)	0.003 **	
		Contralesional	6.63 (2.19)	6.86 (2.82)	6.14 (3.20)	0.44	
	Control	left	7.08 (0.66)				0.0001 *
		right	6.92 (2.14)				0.43
Lactate (mmol/L)	Patient	Ipsilesional	0.90 (1.11)	1.77 (1.61)	2.03 (1.49)	0.001 **	
		Contralesional	0.66 (1.13)	0.70 (1.16)	0.72 (0.67)	0.79	
	Control	left	0.47 (0.70)				0.27
		right	0.30 (0.40)				0.32
CBF (mL/100 g/min)	Patient	Ipsilesional	22.72 (8.18)	31.84 (4.75)	31.04 (4.18)	0.004 **	
		Contralesional	32.93 (8.47)	36.10 (4.82)	32.17 (8.32)	0.58	
	Control	left	36.81 (6.77)				0.004 **
		right	38.64 (7.95)				0.25
**Clinical assessments (patients only)**			**Baseline**	**6 weeks**	**12 weeks**	***p*-value (longitudinal change in patients)**	
Fugl–Meyer			43.3 (30.0)	74.2 (32.2)	78.3 (30.1)	0.009 **	

^§^ For the comparison of concentrations between patients (at baseline) and controls, the baseline ipsilesional thalamic concentrations in patients were compared with left thalamic concentrations in controls * *p* < 0.05, ** *p* < 0.01.

## References

[1] Duncan P.W., Zorowitz R., Bates B., Choi J.Y., Glasberg J.J., Graham G.D., Katz R.C., Lamberty K., Reker D. (2005). Management of Adult Stroke Rehabilitation Care: A clinical practice guideline. Stroke.

[2] Cramer S.C. (2008). Repairing the human brain after stroke: I. Mechanisms of spontaneous recovery. Ann. Neurol..

[3] Zhang Z.G., Chopp M. (2009). Neurorestorative therapies for stroke: Underlying mechanisms and translation to the clinic. Lancet Neurol..

[4] Sztriha L.K., O’Gorman R.L., Modo M., Barker G.J., Williams S.C.R., Kalra L. (2012). Monitoring brain repair in stroke using advanced magnetic resonance imaging. Stroke.

[5] Hervé D., Molko N., Pappata S., Buffon F., LeBihan D., Bousser M.G., Chabriat H. (2005). Longitudinal thalamic diffusion changes after middle cerebral artery infarcts. J. Neurol. Neurosurg. Psychiatry.

[6] Munoz Maniega S., Cvoro V., Chappell F.M., Armitage P.A., Marshall I., Bastin M.E., Wardlaw J.M. (2008). Changes in NAA and lactate following ischemic stroke: A serial MR spectroscopic imaging study. Neurology.

[7] Li C., Ling X., Liu S., Xu A., Zhang Y., Xing S., Pei Z., Zeng J. (2011). Early detection of secondary damage in ipsilateral thalamus after acute infarction at unilateral corona radiata by diffusion tensor imaging and magnetic resonance spectroscopy. BMC Neurol..

[8] Gerhard A., Schwarz J., Myers R., Wise R., Banati R.B. (2005). Evolution of microglial activation in patients after ischemic stroke: A [11 C](R)-PK11195 PET study. Neuroimage.

[9] Zhu H., Barker P.B. (2011). MR Spectroscopy and Spectroscopic Imaging of the Brain. Methods Mol. Biol..

[10] Rae C.D. (2014). A Guide to the Metabolic Pathways and Function of Metabolites Observed in Human Brain 1H Magnetic Resonance Spectra. Neurochem. Res..

[11] Rigotti D.J., Inglese M., Gonen O. (2007). Whole-brain N-acetylaspartate as a surrogate marker of neuronal damage in diffuse neurologic disorders. Am. J. Neuroradiol..

[12] Petroff O.A., Graham G.D., Blamire A.M., al-Rayess M., Rothman D.L., Fayad P.B., Brass L.M., Shulman R.G., Prichard J.W. (1992). Spectroscopic imaging of stroke in humans: Histopathology correlates of spectral changes. Neurology.

[13] Font M.A., Arboix A., Krupinski J. (2010). Angiogenesis, neurogenesis and neuroplasticity in ischemic stroke. Curr. Cardiol. Rev..

[14] Zaharchuk G. (2014). Arterial spin labeled perfusion imaging in acute ischemic stroke. Stroke.

[15] Walker P.M., Ben Salem D., Lalande A., Giroud M., Brunotte F. (2004). Time course of NAA T2 and ADC(w) in ischaemic stroke patients: H-1 MRS imaging and diffusion weighted MRI. J. Neurol. Sci..

[16] Wahlund L.O., Barkhof F., Fazekas F., Bronge L., Augustin M., Sjogren M., Wallin A., Adèr H., Leys D., Pantoni L. (2001). A New Rating Scale for Age-Related White Matter Changes Applicable to MRI and CT. Stroke.

[17] Ward N.S., Brown M.M., Thompson A.J., Frackowiak R.S. (2003). Neural correlates of motor recovery after stroke: A longitudinal FMRI study. Brain.

[18] Provencher S.W. (2001). Automatic quantitation of localized in vivo 1H spectra with LC Model. NMR Biomed..

[19] Dai W., Garcia D., de Bazelaire C., Alsop D.C. (2008). Continuous flow-driven inversion for arterial spin labeling using pulsed radio frequency and gradient fields. Magn. Reason. Med..

[20] Gladstone D.J., Danells C.J., Black S.E. (2002). The fugl-meyer assessment of motor recovery after stroke: A critical review of its measurement properties. Neurorehabil. Neural Repair.

[21] Rehabilitation Measures Database. http://www.rehabmeasures.org/Lists/RehabMeasures/DispForm.aspx?ID=889.

[22] White I.R., Royston P., Wood A.M. (2011). Multiple imputation using chained equations: Issues and guidance for practice. Stat. Med..

[23] Wardlaw J.M., Dennis M.S., Lindley R.I., Warlow C.P., Sandercock P.A.G., Sellar R. (1993). Does early reperfusion of a cerebral infarct influence cerebral infarct swelling in the acute stage or the final clinical outcome?. Cerebrovasc. Dis..

[24] Sijens P.E., den Heijer T., de Leeuw F.E., de Groot J.C., Acheten E., Heijboer R.J., Hofman A., Breteler M.M., Oudkerk M. (2001). MR spectroscopy detection of lactate and lipid signals in the brains of healthy elderly people. Eur. Radiol..

[25] Walter H.L., Walberer M., Rueger M.A., Backes H., Wiedermann D., Hoehn M., Neumaier B., Graf R., Fink G.R., Schroeter M. (2015). In vivo analysis of neuroinflammation in the late chronic phase after experimental stroke. Neuroscience.

[26] Tsai G., Coyle J.T. (1995). N-acetylaspartate in neuropsychiatric disorders. Prog. Neurobiol..

[27] Kirov I.I., Tal A., Babb J.S., Herbert J., Gonen O. (2013). Serial proton MR spectroscopy of gray and white matter in relapsing-remitting MS. Neurology.

[28] O’Gorman R.L., Siddiqui A., Alsop D.C., Jarosz J.M. (2010). Perfusion MRI demonstrates crossed-cerebellar diaschisis in sickle cell disease. Pediatr. Neurol..

[29] Chu W.J., Mason G.F., Pan J.W., Hetherington H.P., Liu H.G., San Pedro E.C., Mountz J.M. (2002). Regional cerebral blood flow and magnetic resonance spectroscopic imaging findings in diaschisis from stroke. Stroke.

[30] Carrera E., Tononi G. (2014). Diaschisis: Past, Present, Future. Brain.

[31] Craciunas S.C., Brooks W.M., Nudo R.J., Popescu E.A., Choi I.Y., Lee P., Yeh H.W., Savage C.R., Cirstea C.M. (2013). Motor and premotor cortices in subcortical stroke: Proton magnetic resonance spectroscopy measures and arm motor impairment. Neurorehabil. Neural Repair.

[32] Bivard A., Krishnamurthy V., Stanwell P., Yassi N., Spratt N.J., Nilsson M., Levi C.R., Davis S., Parsons M.W. (2014). Spectroscopy of reperfused tissue after stroke reveals heightened metabolism in patients with good clinical outcomes. J. Cereb. Blood Flow Metab..

